# Histological Distinction between the Granular and Nongranular Types of Laterally Spreading Tumors of the Colorectum

**DOI:** 10.1155/2014/153935

**Published:** 2014-08-11

**Authors:** Shingo P. Goto, Naoto Sakamoto, Hiroyuki Mitomi, Takashi Murakami, Hideaki Ritsuno, Hiroya Ueyama, Kenshi Matsumoto, Tomoyoshi Shibuya, Taro Osada, Akihito Nagahara, Tatsuo Ogihara, Takashi Yao, Sumio Watanabe

**Affiliations:** ^1^Department of Gastroenterology, Juntendo University School of Medicine, 2-1-1 Hongo, Bunkyo-ku, Tokyo 113-8421, Japan; ^2^Department of Human Pathology, Juntendo University School of Medicine, 2-1-1 Hongo, Bunkyo-ku, Tokyo 113-8421, Japan

## Abstract

Colorectal laterally spreading tumors (LSTs), which are classified into granular (LST-G) and nongranular (LST-NG) types, are a good indication for endoscopic treatment. In practice, the nongranular type is more difficult to remove endoscopically than the granular type. It might be assumed that some histological differences exist between these subtypes. The objective of this study was to analyze histological features of laterally spreading tumors and compare between the granular and the nongranular types. A total of 32 cases of LSTs resected endoscopically being intramucosal tumors with no previous treatment were analyzed. The disposition of the muscularis mucosae, the vascular density, and the degree of fibrosis of the submucosal layer were determined. The outline of the muscularis mucosae in LST-NG was almost flat, but that of LST-G was wavy. The submucosal vascular density was significantly greater in the LST-NGs (61.4 ± 24.3/mm^2^) than in the LST-Gs (43 ± 22.4/mm^2^; *P* = 0.033). There was no clear difference in the degree of submucosal fibrosis between the subtypes. A flat disposition of the muscularis mucosae and a more densely vascularized submucosal layer were characteristics of LST-NGs compared to the LST-Gs. These findings may play a role when performing the endoscopic resection of LSTs.

## 1. Introduction 

**Figure 1:**
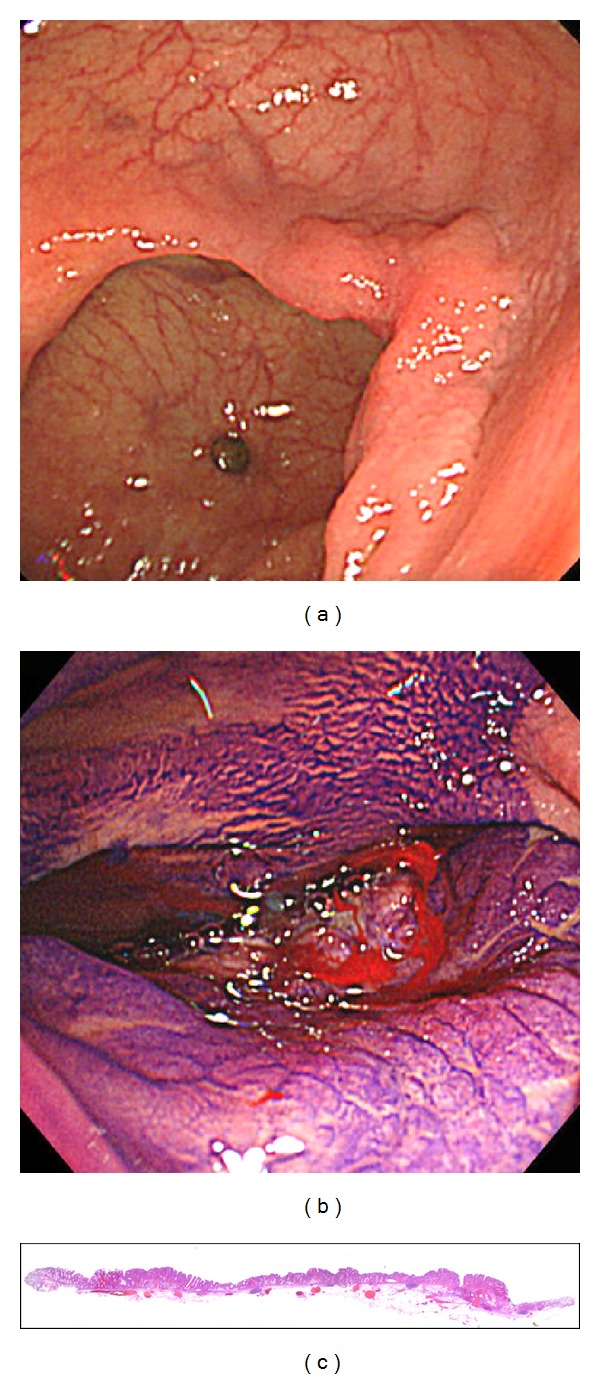
(a) A typical case of a laterally spreading tumor of the nongranular type (LST-NG) located in the cecum. (b) Despite any previous manipulation, the lesion showed a nonlifting sign. (c) The lesion removed en bloc by endoscopic submucosal dissection was found to be an intramucosal carcinoma.

Removing colorectal adenomas is effective in reducing the incidence of colorectal cancer [1]. Most adenomas are protruded and many are pedunculated; thus, they are easily removed through simple snare polypectomy. Sessile or nonpolypoid neoplasms known as laterally spreading tumors (LSTs) are challenging to remove endoscopically, requiring a more advanced technique such as endoscopic mucosal resection (EMR) or endoscopic submucosal dissection (ESD). The LST, firstly described by Kudo, is a colorectal neoplasm characterized by a horizontally extending growth pattern with a relatively low vertical axis [2, 3]. By definition LSTs are larger than 10 mm in diameter and are classified into two types, the granular (LST-G) and nongranular (LST-NG) types. In turn, the LST-G is subclassified into homogeneous and nodular mixed types and the LST-NG into flat elevated and pseudodepressed types. The LST-NGs have a higher malignant potential than LST-Gs [4] and thus are considered a good indication for ESD to avoid unintended piecemeal resections [5]. The lifting [6] of the lesion following the injection of a solution into the submucosal layer is a crucial factor during endoscopic resection. In practice, the lifting characteristics of LST-Gs are adequate for a safe endoscopic resection, but those of LST-NGs are usually unsuitable even for noninvasive cases, making resection by EMR difficult or even impossible (Figure 1). Although the effectiveness of ESD to achieve en bloc resection of LSTs has been demonstrated [7], submucosal dissection of LST-NGs seems laborious, taking a longer operative time than for LST-Gs [8]. Submucosal fibrosis secondary to biopsy and the thickness of the normal submucosal layer beneath the lesion can influence the lifting condition [9, 10]. Furthermore, there seems to be a difference in the vascular supply according to the macroscopic type of polyp [11]. In this study we therefore focused on some histological features of LSTs that may distinguish LST-NGs and LST-Gs. We particularly aimed to analyze the disposition of the muscularis mucosae, submucosal vascular density, and the degree of submucosal fibrosis and compare between both types.

## 2. Materials and Methods

### 2.1. Case Selection

Cases were selected from patients treated at Juntendo University Hospital, Department of Gastroenterology from January 2008 to July 2012. From a total of 247 LSTs resected endoscopically, 16 representative cases for each type of LST (classified by 3 endoscopists examining endoscopy photos) that comply with the following criteria were randomly selected: pathologically adenoma or intramucosal carcinoma, ≥10 mm in size, traceable and uninterrupted muscularis mucosa, and having 250 *μ*m or more appraisable submucosal layer along with the tumor. Furthermore, all the lesions had to be resected in one piece. In order to exclude known factors that may influence the characteristics of the submucosal layer, the neoplasms invading the submucosal or deeper layer, recurrent lesions, lesions with previous manipulations such as biopsy or submucosal injection in an attempted excision, and patients with inflammatory bowel disease were excluded. All specimens were stretched as evenly as possible and pinned onto cardboard immediately upon removal before 10% formalin fixation. Medical records and hematoxylin and eosin (H&E) stained slides of all cases were reviewed for the selection. The paraffin block with the largest amount of tumor tissue was chosen from each case and wascut into 3.5 *μ*m thin slices for immunohistochemical and special stainings.

### 2.2. Analysis of the Disposition of the Muscularis Mucosae (Figure 2)

Histologic slides were prepared by staining with the immunohistochemical marker alpha-smooth muscle actin (*α*-SMA) to delineate the muscularis mucosae. Antibodies used for this study are listed in Table 1. The entire slide was scanned and digitalized using the Virtual Slide System VS-100 (Olympus) and analyzed on computer software VS-ASW (Olympus). Making use of the software's measuring tool, the length of the lesion and of the muscularis mucosae were determined. The length of the lesion itself was measured drawing a beeline from end to end of the neoplastic area. In turn, the length of the muscularis mucosae was measured tracing a “freehand line” over the area outlined by the specific staining mentioned above. The ratios between the two lengths were obtained in order to numerically represent the unevenness of the muscularis mucosae.

### 2.3. Estimating Submucosal Vascular Density (Figure 3)

Serial sections were subjected to H&E, CD31, CD34, factor VIII, D2-40, Duffy antigen/receptor for chemokines (DARC), and double staining with factor VIII and Elastica van Gieson (EVG). Serial pictures of the entire lesion including 250 *μ*m of the submucosal layer for each stain were taken using a digital microscope camera (Olympus DP73) at 40X magnification. The pictures were analyzed on computer by endoscopists and expert pathologists. Density was calculated by identifying and counting all the arterioles and venules located beneath the neoplastic area, using mostly the sections stained with factor VIII + EVG, and expressed as vessels/square millimeters (mm²). Contiguous vessels were considered independent if clear continuity was not observed, and they were counted individually. Those vessels having the tunica intima stained by factor VIII and any of the following characteristics were counted as arterioles: wall thickness approximately equal to half the diameter of the lumen, thin internal elastic lamina (stained black), and thick tunica media (stained yellow). Structures with the tunica intima stained with factor VIII, as with arterioles, but with the wall thickness less than half the diameter of the lumen, were considered to be venules. Vessels less than 5 *μ*m and capillaries and lymphatic vessels were not counted.

### 2.4. Submucosal Fibrosis of LST

For the assessment of histological fibrosis 3.5 *μ*m sections were stained with Masson's Trichrome according to the standard protocol. The entire lesion was scanned using a digital microscope camera at 4X magnification and was examined on computer. The distribution of fibrotic areas did not seem uniform within the same lesion, and considering that there is no well-established method to classify histologically the degree of submucosal fibrosis for colorectal lesions, the method that was reported for gastric lesions was adopted [12]. This system takes into account the intensity and extent of the fibrotic areas. For intensity, 0 was designated for a mostly white or honeycomb-like appearance, 2 for dense staining with blue with almost no white interstice, and 1 was intermediate between 0 and 2. Regarding the extent of the fibrosis, scores were assigned as follows: 0 (0%–10%), 1 (11%–50%), and 2 (51%–100%) according to the proportion of the total area that was stained. The final degree of histologic fibrosis was obtained as the product of each intensity designation (0~2) with its respective designation of extent (0~2), resulting in 0 to 5 points. Classification was pF0 (0, 1 point): no fibrosis; pF1 (2, 3 points): mild fibrosis; and pF2 (4, 5 points): severe fibrosis (Figure 4).

Endoscopically, submucosal fibrosis was assessed by analyzing the digital pictures taken during the procedure and was classified as eF0: no fibrosis; eF1: mild fibrosis; and eF2: severe fibrosis as was previously reported [13]. Since this classification is not commonly used in our institution each case was categorized by three expert endoscopists independently. In cases of disagreement the majority rule was applied.

Statistical analysis was carried out using the chi-squared test and Student's *t*-test, and *P* values of 0.05 were considered as significant.

## 3. Results

Clinical and pathological features of the cases selected for this study are shown in Table 2. Among the 16 lesions for each type of LST, there were 9 homogeneous and 7 nodular mixed types for LST-G and 14 pseudodepressed and 2 flat elevated types for LST-NG. The major axis of LST-G on average (SD) was 30.5 (6.4) mm and was 23.4 (6.7) mm for LST-NG, differing significantly (*P* < 0.01). Except for 5 cases of LST-G and 1 case of LST-NG located in the rectum, most of the lesions were found in the colon. Pathologically, all of the 13 cases of adenomas for LST-NG were the tubular type, whereas for LST-G 6 out of 10 were the tubular type, and the rest was tubulovillous adenomas (*P* = 0.012). ESD was the preferred technique for removal of both types of LSTs. An EMR with the circumferential mucosal incision technique was required for 3 cases of LST-NG.

### 3.1. Length of Tumor and Muscularis Mucosae (Table 3)

**Figure 2:**
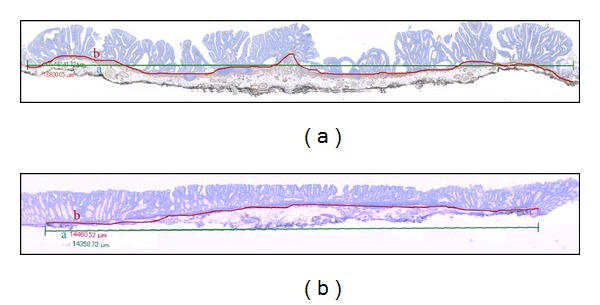
Analysis of the disposition of the muscularis mucosae of laterally spreading tumors (LST). The length of the lesion in a straight line (a: green line) and the length of the muscularis mucosa (MM) (b: red line) were measured digitally. (a) The irregular disposition of the MM of LST-granular type was reflected in greater ratios between both measures (b/a). (b) In contrast the MM of LST-NGs were flat as both lengths (a, b) were nearly similar.

The mean (SD) length of LST-Gs measured on a straight line was 17.28 mm (4.33) and 13.82 mm (2.84) for LST-NGs. However, the mean length of the muscularis mucosae was greater in both types (LST-G 18.48 ± 4.93 mm versus LST-NG 14.06 ± 2.95 mm). The mean ratio between the length of the muscularis mucosae and the length of the lesion was more pronounced for LST-G (1.067 ± 0.073, *P* = 0.01), probably as a result of the presence of intraepithelial papilla-like submucosal protrusions under the nodules together with the muscularis mucosae (Figure 2(a)). In contrast, the muscularis mucosae of LST-NG as outlined by *α*-SMA were almost straight, with the mean ratio between both measurements 1.016 ± 0.010 (Figure 2(b)).

### 3.2. Submucosal Vascular Density of LST (Table 4)

**Figure 3:**
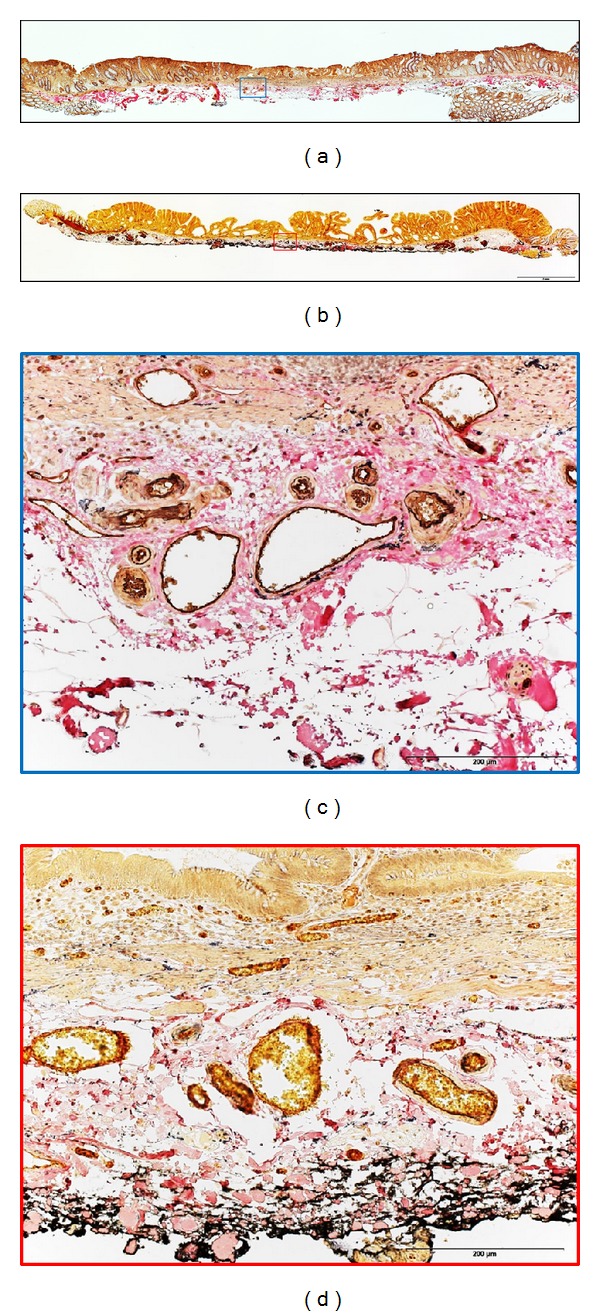
Estimation of submucosal vascular density of laterally spreading tumors (LSTs): all the vessels ≥5 *μ*m in diameter were counted and arterioles and venules were distinguished. (a) A typical case of LST-nongranular (LST-NG) type with higher vascular density (56.3 vessels/mm²) than (b) a LST-granular (LST-G) type with 31.7 vessels/mm². (c) The double staining with Factor VIII + Elastica van Gieson has facilitated the identification of arterioles and venules for LST-NG and for (d) LST-G, original magnification ×20.

**Figure 4:**
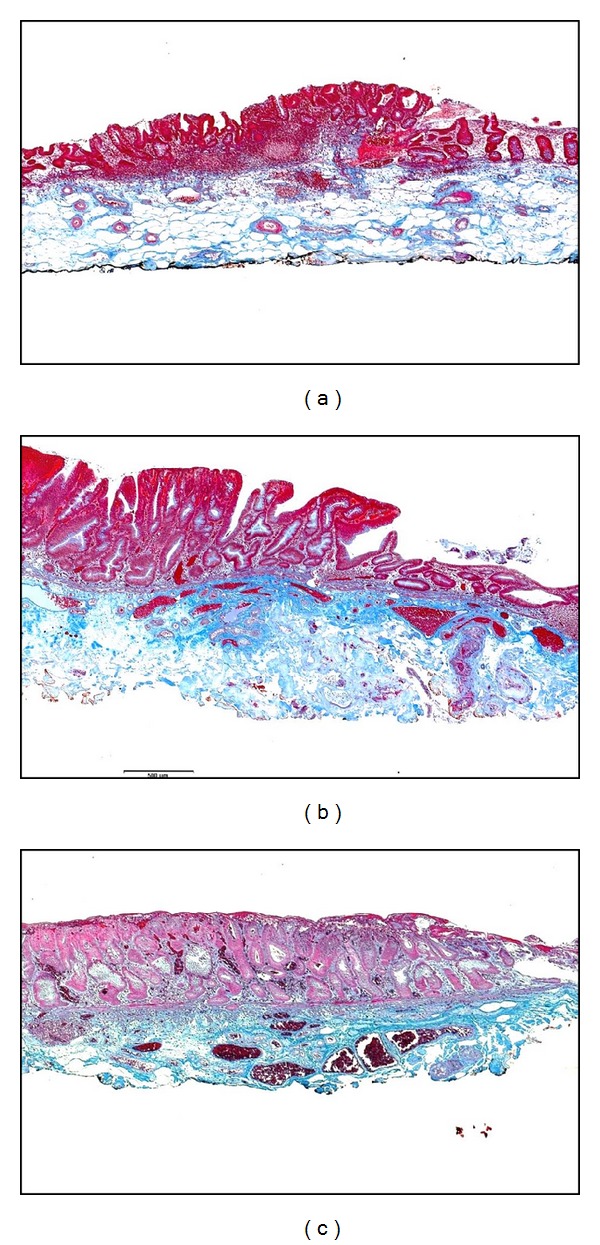
Sections of laterally spreading tumor-nongranular (LST-NG) type with different degrees of submucosal fibrosis. (a) F0: no fibrosis, honeycomb-like appearance throughout; (b) F1: mild fibrosis, intensity of staining was high in *≺*50% of the sample; (c) F2: severe fibrosis, intensity of staining was high almost throughout (Masson's trichrome stain, original magnification ×10).

In the preliminary examination of 10 cases, double staining with factor VIII and EVG (Figure 3) was considered the most appropriate to identify the vessels, distinguishing at the same time arterioles and venules. Therefore all cases were evaluated based on this staining. The mean density of vessels (arterioles + venules) in the submucosal layer was significantly higher in LST-NG (61.4 ± 24.3/mm²) than in LST-G (43 ± 22.4/mm²) (*P* = 0.033). This difference was at the expense of the higher density of arterioles for LST-NG (32.7 ± 11.9/mm² versus LST-G 18.3 ± 8.1/mm²), since the densities of venules were similar between the two types.

### 3.3. Submucosal Fibrosis (Table 5)

Endoscopically, 5 of the 16 LST-G lesions and 6 of the 16 LST-NG lesions were classified as eF1 (mild fibrosis). The remaining lesions (LST-G 11/16, LST-NG 10/16) had no fibrosis (eF0). However, on histological examination, 2 lesions in each type of LST were categorized as pF2 (severe fibrosis); furthermore, there were more pF1 lesions among both types (LST-G 7/16, LST-NG 9/16). Although there were some differences with respect to the degree of endoscopic and histological fibrosis, there was no clear disparity in proportions that were observed between LST-G and LST-NG (*P* values for endoscopic and pathological fibrosis were 0.709 and 0.747, resp., *χ* ^2^ test).

## 4. Discussion 

**Figure 5:**
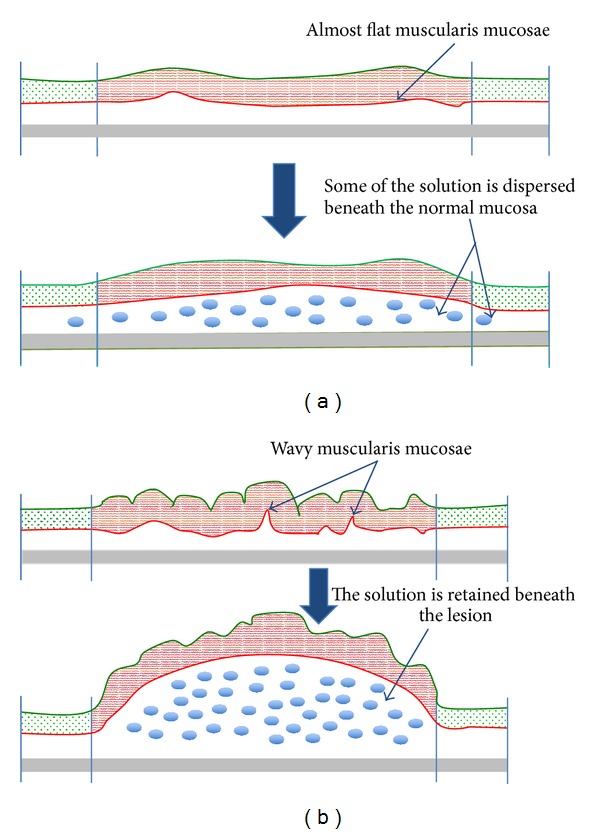
Representation of laterally spreading tumors before and after the injection of a solution into the submucosal layer. (a) The almost flat muscularis mucosae of LST-NGs may lead to disperse the solution, elevating not only the lesion but also the surrounding mucosa. (b) In contrast, the wavy disposition of the muscularis mucosae of LST-Gs may facilitate to retain the solution beneath the lesion, elevating consequently the lesion and making propitious for EMR or ESD.

In the present study we demonstrated histological differences between the two types of LSTs. Firstly, the muscularis mucosae of LST-NGs were almost flatwithout the submucosal protrusions that were observed in the majority of LST-Gs (Figure 5). Moreover, the submucosal layer of LST-NGs was more densely vascularized, while after evaluating the entire lesion no evident differences in the degree of submucosal fibrosis was observed between the two types of LSTs.

This is the first study to analyze the disposition of the muscularis mucosae of LSTs. The degree of elevation or lifting of the lesion during EMR or ESD is of utmost importance to achieve safe and en bloc resections. According to previous reports, when the carcinoma invades deeply into the submucosal layer (sm2 or sm3), the lesion becomes “nonlifted,” in which only the surrounding mucosa becomes elevated [6, 10, 14]. Furthermore, it seems that, rather than the presence of malignant tissue within the submucosal layer, the distance between the lower limit of the carcinoma and the resection line or the muscularis propria affects to a greater extent the degree of elevation [10]. As suggested in this report, it appears that approximately 1000 *μ*m of normal submucosal thickness beneath the lesion is needed for an appropriate elevation. For intramucosal lesions, as in our cases, the muscularis mucosae may work as the variable limit of the extent of the submucosal layer, since the muscularis propria of the bowel seems uniform and remains relatively firm during endoscopic resection [15]. In this sense, we found that while the muscularis mucosae of LST-Gs was irregular due to the presence of submucosal protrusions it was almost lineal in LST-NGs. The wavy disposition of the muscularis mucosae and the seemingly more spacious submucosal layer of LST-Gs may favor the accumulation of the injected solution beneath the lesion, increasing at the same time the thickness of the submucosal layer (Figure 5(b)). Although no study has compared the thickness of the submucosal layer of LSTs, in coincidence with our experience, it was suggested that the thinner submucosal layer of the LST-NG could make those more difficult to resect [8]. The tumor's weight in conjunction with peristalsis could elongate the muscularis mucosae, since submucosal protrusions were observed in coincidence with the granular portions of LST-G.

Fibrosis caused extrinsically, for example, by cold biopsy, is well known to have a negative effect, making the resection of colorectal lesions more complicated [9]. However, regarding intrinsic or “de novo” fibrosis of LSTs little is known with certainty. Concurring with some experts, in our experience “de novo” fibrosis of the submucosal layer is frequently observed for LST-NG [4, 8]. To clarify this issue, we selected only naïve lesions, that is, those without previous manipulations such as biopsy or any predictable factors that could affect the characteristics of the submucosal layer. Adopting the classification system for gastric tumors, we did find histologically “de novo” fibrosis of the submucosal layer for LST-NG (2 pF2 and 9 pF1 of 16 lesions) and also for LST-G (2 pF2 and 7 pF1 of 16 lesions). In one previous report, which examined all kinds of tumors, including recurrent and invasive ones, severe fibrosis (eF2) was more frequent for the nodular mixed-type of LST-G, but they found no difference between LST-Gs and LST-NGs [13]. Although that study included lesions with fibrosis possibly caused by extrinsic factors, our examination found similar results with respect to the proportion of lesions with fibrosis between the two types of LSTs. Besides obvious cases, the occurrence of submucosal fibrosis seems more likely in the presence of ulceration, tumor size ≥30 mm, submucosal invasion, and depressed-types of gastric tumors [12, 16]. In our cases, even though the mean size of LST-Gs was significantly larger than that of LST-NGs (30.5 mm versus 23.4 mm) and most LST-NGs were of the pseudodepressed type, we found no difference in the frequency of fibrosis between the two types of LSTs. Previous reports also showed that submucosal fibrosis by any cause in gastric and colorectal tumors is closely related to a longer procedure time and risk of complications, such as perforation and bleeding [12, 17]. As to the discordance in the number of endoscopic and histological fibrosis, that discordancemight be due to observations of digitally scanned images of the entire lesions, making possible a more complete overall determination of the histological fibrosis. In contrast, the endoscopic submucosal fibrosis, although was focused on the most fibrotic areas, was based on a series of intermittently taken pictures during the ESD, not to mention the role of the subjectivity, particularly for discerning eF0 and eF1, since eF2 lesions were clearly different.

Relatively large vessels seemed to concentrate under the protruded part of the tumors as shown in a previous study [11]. We found that the granular portion of LST-Gs has that pattern of blood supply whereas LST-NGs were supplied by more uniformly distributed small caliber vessels. Even without magnifying endoscopy, large venules and arterioles are easily identified and cauterized by switching to a coagulating device, but small ones could be cut inadvertently during ESD. Whether the vessels are identified or not, the more richly vascularized submucosal layer of LST-NG may require a greater operative time for cauterizing each vessel and also impose a greater risk of microhemorrhage.

This study had limitations. It was a retrospective study. Also, the number of cases was limited because they were selected under strict inclusion criteria. This may have influenced to demonstrate differences in the occurrence of “de novo” fibrosis between both types of LSTs contrary to some views that “de novo” fibrosis is frequent in LST-NGs. There are two types of LST-NGs, but in our cases these were mostly the pseudodepressed type (14/16), which may have led to a selection bias. This study in its entirety was based on endoscopically resected specimens; therefore, the aspect of the submucosal layer after a submucosal cushion was made would be different from its natural state. Although difficult to obtain due to the popularization and improvements in endoscopic techniques, surgically resected specimens of LSTs would be more appropriate for this purpose and also to compare the thickness of the submucosal layer between LST-Gs and LST-NGs.

In conclusion, we found that when it comes to intramucosal and nonrecurrent tumors, two intrinsic features clearly differed between LST-NGs and LST-Gs. The almost flat disposition of the muscularis mucosae and more densely vascularized submucosa in LST-NGs may play a role when performing the endoscopic resection. However, a prospective comparative study between the two types of LST with more cases is required to demonstrate the association between these features and the outcomes of ESD and EMR.

## Figures and Tables

**Table 1:** Antibodies used in the study.

Antibody	Dilution	Source	Clone	Incubation
Monoclonal mouse anti-human CD31	1/40	DakoCytomation; Glostrup, Denmark	JC70A	60 min
Monoclonal mouse anti-human CD34	1/50	DakoCytomation; Glostrup, Denmark	QBEnd 10	60 min
Monoclonal mouse anti-human D2-40	1/100	DakoCytomation; Glostrup, Denmark	D2-40	60 min
Polyclonal rabbit anti-human Von Willebrand factor	1/300	DakoCytomation; Glostrup, Denmark		60 min
Polyclonal goat anti-human DARC	1/200	Abcam; Cambridge, MA, USA		60 min
Smooth muscle actin	1/200	DakoCytomation; Glostrup, Denmark	1A4	Overnight

**Table 2:** Clinicopathological features of patients with laterally spreading tumor included in this study (*n* = 32).

Subtypes of LST	LST-G (16)	LST-NG (16)	*P*
	9 Homogeneous/7 nodular mixed	14 Pseudodepressed/2 flat-elevated	
Gender (male/female)	6/10	11/5	0.076
Mean age (years, mean ± SD)	67.9 ± 7.8	71.7 ± 6.8	0.153
Mean tumor size (mm, mean ± SD)	30.5 ± 6.4	23.4 ± 6.7	<0.01
Tumor location (Colon/Rectum)	11/5	15/1	0.070
Resection method (ESD/EMR)	14/2	13/3	0.626
Pathology (adenoma/intramucosal carcinoma)	10/6	13/3	0.238
Adenoma (tubular/tubulovillous)	6/4	13/0	0.012
Carcinoma (with adenoma/without adenoma component)	3/3	1/2	0.635

**Table 3:** Length of tumor and muscularis mucosae of laterally spreading tumor-granular (LST-G) and laterally spreading tumor-nongranular (LST-NG) types.

	LST-G (16)	LST-NG (16)	*P* value
Length of lesion^a^ (mm, mean ± SD)	17.28 ± 4.33	13.82 ± 2.84	0.012
Length of muscularis mucosae^b^ (mm, mean ± SD)	18.48 ± 4.93	14.06 ± 2.95	<0.01
Ratio between b and a (b/a) (mean ± SD)	1.067 ± 0.073	1.016 ± 0.010	0.01

**Table 4:** Submucosal vascular density of laterally spreading tumor-granular (LST-G) and laterally spreading tumor-nongranular (LST-NG) types.

	LST-G (16)	LST-NG (16)	*P* value∗
Microvessel density (arterioles + venules/mm^2^, mean ± SD)	43 ± 22.4	61.4 ± 24.3	0.033
Arteriole's density (arteriole/mm^2^, mean ± SD)	18.3 ± 8.1	32.7 ± 11.9	<0.01
Venule's density (venule/mm^2^, mean ± SD)	24.7 ± 18	28.6 ± 17.7	0.537

∗Student's *t*-test.

**Table 5:** Relationships between subtypes of laterally spreading tumor (LST) and degree of fibrosis.

	Degree of endoscopic fibrosis∗		Degree of histological fibrosis∗∗		
	eF0	eF1	pF0	pF1	pF2
LST-G (*n* = 16)	11	5	7	7	2
LST-NG (*n* = 16)	10	6	5	9	2

*P = 0.709; **P = 0.747, chi-square test.

## References

[1] Winawer SJ, Zauber AG, O'Brien MJ (1993). Prevention of colorectal cancer by colonoscopic polypectomy. *The New England Journal of Medicine*.

[2] Kudo S, Kashida H, Tamura T (2000). Colonoscopic diagnosis and management of nonpolypoid early colorectal cancer. *World Journal of Surgery*.

[3] Lambert R, Kudo SE, Vieth M (2009). Pragmatic classification of superficial neoplastic colorectal lesions. *Gastrointestinal Endoscopy*.

[4] Uraoka T, Saito Y, Matsuda T (2006). Endoscopic indications for endoscopic mucosal resection of laterally spreading tumours in the colorectum. *Gut*.

[5] Tanaka S, Tamegai Y, Tsuda S, Saito Y, Yahagi N, Yamano H (2010). Multicenter questionnaire survey on the current situation of colorectal endoscopic submucosal dissection in Japan. *Digestive Endoscopy*.

[6] Kato H, Haga S, Endo S (2001). Lifting of lesions during endoscopic mucosal resection (EMR) of early colorectal cancer: implications for the assessment of resectability. *Endoscopy*.

[7] Nakajima T, Saito Y, Tanaka S (2013). Current status of endoscopic resection strategy for large, early colorectal neoplasia in Japan. *Surgical Endoscopy*.

[8] Toyonaga T, Man-I M, Fujita T (2010). Retrospective study of technical aspects and complications of endoscopic submucosal dissection for laterally spreading tumors of the colorectum. *Endoscopy*.

[9] Han KS, Sohn DK, Choi DH (2008). Prolongation of the period between biopsy and EMR can influence the nonlifting sign in endoscopically resectable colorectal cancers. *Gastrointestinal Endoscopy*.

[10] Ishiguro A, Uno Y, Ishiguro Y, Munakata A, Morita T (1999). Correlation of lifting versus non-lifting and microscopic depth of invasion in early colorectal cancer. *Gastrointestinal Endoscopy*.

[11] Dobrowolski S, Dobosz M, Babicki A, Głowacki J, Nałecz A (2006). Blood supply of colorectal polyps correlates with risk of bleeding after colonoscopic polypectomy. *Gastrointestinal Endoscopy*.

[12] Jeong JY, Oh YH, Yu YH (2012). Does submucosal fibrosis affect the results of endoscopic submucosal dissection of early gastric tumors?. *Gastrointestinal Endoscopy*.

[13] Matsumoto A, Tanaka S, Oba S (2010). Outcome of endoscopic submucosal dissection for colorectal tumors accompanied by fibrosis. *Scandinavian Journal of Gastroenterology*.

[14] Uno Y, Munakata A (1994). The non-lifting sign of invasive colon cancer. *Gastrointestinal Endoscopy*.

[15] Binmoeller KF, Weilert F, Shah J, Bhat Y, Kane S (2012). “Underwater” EMR without submucosal injection for large sessile colorectal polyps (with video). *Gastrointestinal Endoscopy*.

[16] Higashimaya M, Oka S, Tanaka S (2013). Outcome of endoscopic submucosal dissection for gastric neoplasm in relationship to endoscopic classification of submucosal fibrosis. *Gastric Cancer*.

[17] Isomoto H, Nishiyama H, Yamaguchi N (2009). Clinicopathological factors associated with clinical outcomes of endoscopic submucosal dissection for colorectal epithelial neoplasms. *Endoscopy*.

